# Genetic determinants of lactococcal *C2viruses* for host infection and their role in phage evolution

**DOI:** 10.1099/jgv.0.000499

**Published:** 2016-08

**Authors:** Anne M. Millen, Dennis A. Romero

**Affiliations:** DuPont Nutrition and Health, Madison, Wisconsin, USA

**Keywords:** Phage, *Lactococcus*, infection, genetic determinants

## Abstract

*Lactococcus lactis* is an industrial starter culture used for the production of fermented dairy products. Pip (phage infection protein) bacteriophage-insensitive mutant (BIM)* L. lactis* DGCC11032 was isolated following challenge of parental strain DGCC7271 with *C2viruses*. Over a period of industrial use, phages infecting DGCC11032 were isolated from industrial whey samples and identified as *C2viruses*. Although Pip is reported to be the receptor for many *C2viruses* including species type phage c2, a similar cell-membrane-associated protein, YjaE, was recently reported as the receptor for *C2virus* bIL67. Characterization of DGCC7271 BIMs following challenge with phage capable of infecting DGCC11032 identified mutations in *yjaE*, confirming YjaE to be necessary for infection. DGCC7271 YjaE mutants remained sensitive to the phages used to generate *pip* variant DGCC11032, indicating a distinction in host phage determinants. We will refer to *C2viruses* requiring Pip as c2-type and*C2viruses* that require YjaE as bIL67-type. Genomic comparisons of two c2-type phages unable to infect *pip* mutant DGCC11032 and four bIL67-type phages isolated on DGCC11032 confirmed the segregation of each group based on resemblance to prototypical phages c2 and bIL67, respectively. The distinguishing feature is linked to three contiguous late-expressed genes: l14–15–16 (c2) and ORF34–35–36 (bIL67). Phage recombinants in which the c2-like l14–15–16 homologue gene set was exchanged with corresponding bIL67 genes ORF34–35–36 were capable of infecting a *pip* mutated host. Together, these results correlate the phage genes corresponding to *l14–15–16* (c2) and ORF34–35–36 (bIL67) to host lactococcal phage determinants Pip and YjaE, respectively.

## Introduction

Highly specialized strains of *Lactococcus lactis* are used as starter cultures for commercial dairy fermentations. Their long-term utility is limited by persistent assault from lytic bacteriophages which are inherent to the commercial fermentation process and are a major cause of fermentation failure and product defects. Therefore, there has been a significant amount of research devoted to lactococcal phage biology, genetics and host interactions (Garneau & Moineau, 2011; Mahony, 2012). Lactococcal phages have been classified into 10 groups based on morphology, DNA homology and more recent genetic characterization using available genome sequences (Jarvis, 1984; Deveau *et al.*, 2006). In particular, phages from the c2, 936 and P335 groups have been consistently found to be problematic in the fermented dairy products industry. *C2virus* (genus as recognized by the International Committee on Taxonomy of Viruses, ICTV) isolates characterized to date have been shown to share DNA homology but to have diverse host ranges (Lubbers *et al.*, 1995). The genus *C2virus *is represented by two species, the eponymous type phage c2 and bIL67, which were among the earliest lactococcal phage genomes sequenced and are the only two *C2virus *species with complete published genome sequences (Lubbers *et al.*, 1995; Schouler *et al.*, 1994). Both genomes are approximately 22 kb in size and are 80 % identical at the nucleotide level with a region of relatively low conservation consisting of structural genes believed to be involved in host-range determination (Lubbers *et al.*, 1995).

This poorly conserved region consists of three late expressed genes, *l14*,* l15* and *l16* in c2 which correspond to genes ORF34, ORF35 and ORF36 in bIL67, respectively. The putative gene products (gp) gpl14 (NP_043562) and gp34 (NP_042311) are annotated as hypothetical proteins. [Note that in NC_001629.1 (Schouler *et al.*, 1994); bIL67 genes ORF34–35–36 are annotated as gp04–03–02. For ease of clarity we will refer to the gene products using their respective ORF gene numbers.] gpl15 (NP_043563) is a minor structural protein, while hypothetical protein gp35 (AAO49840) is proposed to be a host-determinant protein (Stuer-Lauridsen *et al.*, 2003). gpl15/gp35 show conservation at the N termini and to a lesser degree the C-terminal ends while the middle is variable and likely to be involved in host recognition. It has been shown that recombinant bIL67 isolates which maintained the 5′ and 3′ ends of gene ORF35 but exchanged the middle section for that of phage CHL92 ORF2 acquired the host range of CHL92. Adsorption patterns of these hybrid bIL67 isolates were equivalent to those of CHL92 (Stuer-Lauridsen *et al.*, 2003). Lastly, gpl16 (NP_043564) is a minor structural protein while hypothetical protein gp36 (NP_042309) is possibly a collar protein (AAD20610).

In the infection process, lactococcal phages including *C2viruses* initially recognize carbohydrate receptors to bind to the host cell wall (for recent reviews see Ainsworth *et al.*, 2014; Chapot-Chartier, 2014; Mahony *et al.*, 2016; McCabe *et al.*, 2015). More extensive studies have been performed on carbohydrate receptors of the 936 and P335 type phages while studies of *C2viruses *have focused on a cell membrane protein secondary receptor designated Pip (phage infection protein) (Valyasevi *et al.*, 1991; Monteville *et al.*, 1994). For phage c2, binding to carbohydrate receptors is reversible, and interaction with Pip results in irreversible binding followed by phage DNA ejection (Monteville *et al.*, 1994; Geller *et al.*, 1993). The lactococcal Pip protein, first described in *L. lactis* C2 (GenBank accession number L14679), is a 901 aa membrane-spanning protein encoded by a 2706-bp gene (Geller *et al.*, 1993) with homologues in most Gram-positive bacteria (Mooney *et al.*, 2006); notably *Bacillus subtilis *orthologue *yueB *(São-José *et al.*, 2004, 2006). It has been reported that inactivation of *pip* virtually eliminates infection by *C2virus *in lactococci without any apparent effect on the host (Garbutt *et al.*, 1997; Kraus & Geller, 1998). Similarly, SPP1 requires YueB to infect *B.* *subtilis*, where the interaction has been extensively studied (Plisson *et al.*, 2007; Jakuytė *et al.*, 2012). In addition to Pip, a second lactococcal protein, designated as YjaE, was shown to be involved in *C2virus *infection in lactococci (Stuer-Lauridsen & Janzen, 2006; Derkx *et al.*, 2014). YjaE is annotated as a hypothetical protein of 799 aa, encoded by a 2400 nt gene, and described as a ‘transmembrane protein, similar to phage infection protein Pip’ in the most recently curated annotation of *L. lactis *IL1403 (accession NC002662). Contrary to the published literature, it was reported that certain *C2viruses* (including bIL67) were unaffected by a mutation in *pip*. These phages, however, were unable to infect *yjaE-*mutated host strains. The basis of this requirement for host Pip or YjaE for infection was not further examined.

In this study we provide corroborating data distinguishing *C2viruses *which require Pip and those which require YjaE for infection and further correlate this distinction to specific phage-encoded genes. Genome comparisons of industrially isolated *C2viruses *that plaqued differentially on *pip- *or *yjaE-*mutated strains, correlated the requirement for *pip* or *yjaE* to the region showing poor conservation between phages bIL67 and c2. The utilization of Pip or YjaE corresponds to genes *l14–15–16 *(c2) and ORF34–35–36 (bIL67), respectively. In addition, the generation of phage–phage recombinants further demonstrates how *in vivo *genetic modular exchange between co-infecting phages evolves phages able to circumvent host barriers for host infection.

**Table 1.** Phage resistance phenotype

		Strain			
		DGCC7271* pip*^+^/*yjaE*^+^	DGCC11032* pip*^–^/*yjaE*^+^	DGCC11785* pip*^+^/*yjaE*^–^	DGCC11572* pip*^–^/*yjaE*^–^
**c2-type phage**	D4412	S	R	S	R
	M5938	S	R	S	R
	M5939	S	R	S	R
	M5940	S	R	S	R
**bIL67-type phage**	D4410	S	S	R	R
	M6162	S	S	R	R
	M6165	S	S	R	R
	M6202	S	S	R	R
	bIL67	R	R	R	R
	M6653	S	S	R	R
	M6654	S	S	R	R

R, Resistant, which indicates an efficiency of plaquing (EOP; plaque count relative to plaque count on replicating host) of <1×10^−8^. S, Sensitive, which indicates an EOP of 1±0.45.

## Results and Discussion

### Phage isolation and host determinants

The lactococcal *pip* gene encodes a membrane-associated protein that has been shown to serve as a receptor for *C2viruses *including phage c2 (Geller *et al.*, 1993). *pip* mutations cause no apparent detrimental effects on cell physiology and have been reported to confer complete resistance to this group of phages. Therefore, *pip *inactivation offers an effective method for the industrial control of *C2viruses* in dairy fermentations. Industrial starter culture *L. lactis* DGCC7271 had become phage-sensitive according to routine whey testing. Three phages, M5938, M5939 and M5940, were isolated from geographically distinct US dairy factories and determined to be *C2virus* by multiplex PCR (data not shown). These phages were used to isolate bacteriophage insensitive mutants (BIMs) of DGCC7271 in a single high-titre phage challenge. A representative BIM, designated DGCC11032, tested fully resistant to these phages and was found to have a 487-bp deletion in its *pip* gene. This deletion is expected to result in a Pip protein which is non-functional as a phage receptor based on the resistance of DGCC11032 to *C2virus* phages. DGCC11032 was also resistant to *C2virus* D4412 (Table 1) which was isolated in Europe 7 years earlier and not used in the phage challenge. DGCC7271 was therefore replaced by DGCC11032 in industrial starter applications.

Subsequent to its use as a commercial dairy starter, virulent phages were observed against DGCC11032 during routine industrial whey testing. Three representative phages attacking DGCC11032 (M6162, M6165 and M6202) were purified from geographically distinct locations in the USA and Canada for further study. The three phages were infective on parent DGCC7271 and *pip *mutant DGCC11032 at equal efficiency (Table 1). Additionally, phage D4410 which was isolated on DGCC7271 in Europe in 2003 was able to infect parent DGCC7271 and *pip *mutant DGCC11032 at equal efficiency (Table 1). These phages, which typed as *C2virus*, must therefore require a host determinant distinct from Pip for infection.

The *yjaE* gene of DGCC11032 was sequenced and found to be intact, which strongly indicated that these new phages were utilizing YjaE. We investigated this probability by conducting single high-titre phage challenges of DGCC7271 with phage M6162 and separately M6165. Ten BIMs generated from each phage challenge were selected, and their *yjaE* genes were sequenced. All 20 BIMs contained a mutation in *yjaE *including frame shifts via single-base insertion (two unique isolates) or deletion (four unique isolates), IS981A insertion (two unique isolates) and single-base-substitution nonsense mutations (four unique isolates). The remaining eight isolates were not unique. In one case the same nonsense mutation was found in a BIM from the M6162 challenge and a BIM from the M6165 challenge. These results confirm the requirement of *yjaE* for infection. One *yjaE *BIM, designated DGCC11785, which has a single-nucleotide deletion (ΔA within a string of A nucleotides at position 186–192 of *yjaE*) resulting in a premature stop codon at amino acid residue 75, was selected for further characterization. DGCC11785 has an intact *pip *gene and is fully sensitive to phage M5938 (Table 1), confirming that *yjaE* is not required for infection by M5938.

For the purpose of this study, we designate *C2viruses* that require YjaE for lactococcal infection as bIL67-type to distinguish them from *C2viruses* that require Pip which we refer to as c2-type (Table 2), reflective of the two species representing the genera. Representative examples of both c2 and bIL67-type industrial phages which are infective on wild-type DGCC7271 were sequenced.

**Table 2.** Bacterial strains, phages and sequencing primers

Strain		Comments	Source (reference)				
*Lactococcus lactis *ssp. *lactis *DGCC7271		Parent	Industrial starter – DuPont Global Culture Collection				
*Lactococcus lactis *ssp. *lactis *DGCC11032		DGCC7271 *pip* mutant	Industrial starter (this study)				
*Lactococcus lactis *ssp. *lactis *DGCC11785		DGCC7271 *yjaE* mutant	Industrial starter (this study)				
*Lactococcus lactis *ssp. *lactis *DGCC11572		DGCC7271 *pip yjaE* double mutant	This study				
*Lactococcus lactis *ssp. *lactis *IL1403		bIL67-propagating host	Provided by S. Moineau: GenBank accession no. NC_002662.1 (Bolotin *et al.*, 2001)				
		
**Phage**	**Propagation host**	**Type**	**Source**	**Genome (bp)**	**ORFs**	**Percentage GC**	**GenBank accession no. (reference)**
M5938	DGCC7271	c2-type	DuPont collection (isolated 2010, USA)	22 240	38	35.9	KX373687
M5939	DGCC7271	c2-type	DuPont collection (isolated 2010, USA)	nd	–	–﻿	–
M5940	DGCC7271	c2-type	DuPont collection (isolated 2010, USA)	nd	–	–	–
D4412	DGCC7271	c2-type	DuPont collection (isolated 2003, Europe)	22 884	40	35.4	KX373686
M6162	DGCC11032	bIL67-type	DuPont collection (isolated 2012, USA)	21 697	38	35.8	KX373688
M6165	DGCC11032	bIL67-type	DuPont collection (isolated 2012, USA)	22 179	37	35.7	KX373689
M6202	DGCC11032	bIL67-type	DuPont collection (isolated 2012, Canada)	22 251	38	35.9	KX373690
D4410	DGCC7271	bIL67-type	DuPont collection (isolated 2003, Europe)	22 825	40	35.7	KX373685
M6653	DGCC11032	bIL67-type	Hybrid M5938 :: bIL67 (this study)	22 157	37	35.9	KX373691
M6654	DGCC11032	bIL67-type	Hybrid M5938 :: bIL67 (this study)	22 350	38	35.9	KX373692
bIL67	IL1403	bIL67-type	Species type	22 195	37	36.0	NC_001629.1 (Schouler *et al.*, 1994)
c2	Not applicable	c2-type	Species type	22 163	39	36.3	NC_001706.1 (Lubbers *et al.*, 1995)
							
**Primers**			**Sequence (5′ – 3′)**				
pipStartF			TGTCCATTAAAAGGAAGCAGTG				
pipEndR			TTTCTTGCACACGTTCTTCG				
YjaE F1			CCATAAAGAAAGGGGAAAAGATG				
YjaE F2			GTGTGCAACAATTATCTGAAGG				
YjaE F3			GAGGTTCAAGTTTAACAAGTGG				
YjaE R			TGTGCTTTCTAGGACAAATCC				

nd, Not determined.

### Phage sequence analysis

**Fig. 1.**
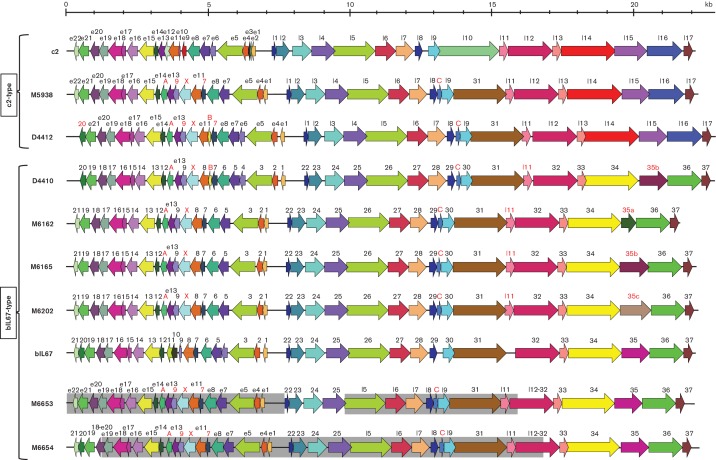
Genome maps of *L. lactis* DGCC7271 *C2viruses* in comparison with species type phages c2 and bIL67. The CDS names as listed in GenBank for phage c2 and bIL67 are depicted above the respective genes in the linear map. Gene names shown in red denote a CDS that was annotated according to the other respective phage type based on an 80 % nucleotide identity and coverage threshold (see text for further details). For hybrid phages M6653 and M6654, which are derived from recombination between bIL67 and M5938, the genome segments originating from parental phage M5938 are shaded in grey.

Phage genome sequences were assembled against phages c2 and bIL67, annotated and CDSs (coding DNA sequence) compared. Overall, the genome size and synteny of the sequenced industrial phage isolates are similar to those of reference *C2virus* species phage c2 and bIL67 (Fig. 1,  Table 2). ORF numbering was assigned based on identities to the corresponding genes of the two respective phages type species. Based on an 80 % nucleotide identity cutoff, each CDS (coloured arrows) was assigned to a gene family with each family depicted as a unique colour. Manual curation was performed to confirm family assignments. The CDS names as listed in GenBank for phage c2 and bIL67 are depicted above the respective genes in the linear maps. For purposes of comparison, genes in each of the industrial phages are labelled according to the corresponding gene names for c2-type (based on c2) and bIL67-type (based on bIL67) as determined by the presence of genes *l14–15–16* or ORF34–35–36, respectively. Gene names shown in red text denote a CDS that was annotated according to the other respective phage type based on an 80 % nucleotide identity and coverage threshold. For purposes of comparison, ORF35 homologues in the industrial phages were likewise named after type phage bIL67 and distinguished by a lower case letter despite having less than 80 % nucleotide identity.

CDS A and B are unique to the industrial phages. CDS C is present in phages c2 (intergenic between *l8l9*) and bIL67 (intergenic between ORF29 and ORF30), but not annotated as a CDS. CDS X is unique to the industrial phages and has identity to lactococcal 936-type phages. Notably, bIL67 ORF9 is smaller than the automated RAST (Rapid Annotation using Subsystem Technology) call for the industrial phage homologues which initiated from an upstream GTG codon; c2 gene *l11* sequence homology is found in bIL67 between ORF31 and ORF32, but not annotated as a CDS. Homology with c2 genes *e2* and *e3* sequences is also found in the corresponding positions in bIL67, D4410 and D4412, but not annotated as a CDS.

In comparing phages c2 and bIL67, Lubbers *et al.* (1995) reported a high level of overall sequence identity with a noted region of lower conservation. The same is observed for the six sequenced industrial phages and is exemplified by the comparison of phages M5938 (c2-type) and M6162 (bIL67-type). The overall nucleotide pairwise identity between M5938 and M6162 is 90.7 %, with approximately the first 17.6 kb being 99.98 % identical. The area of lowest identity (51.9 %) occurs in the region known to be variable among lactococcal *C2virus* phages. This region consists of genes *l14*,* l15* and *l16* in phage c2 which corresponds to ORF34, ORF35 and ORF36 in bIL67. These genes are believed to encode structural proteins and may comprise a structural module for phage evolution (Lubbers *et al.*, 1995; Perrin *et al.*, 1997). At the protein level, Lubbers *et al. *(1995) reported percentage amino acid identities of 43 % (gpl14/gp34), 53 % (gpl15/gp35) and 38 % (gpl16/gp36). A full comparison of pairwise amino acid identity and residue conservation and secondary structure prediction alignments for the respective proteins from all phages in this study are shown in Supplementary Files (available in the online Supplementary Material).

c2-type phages D4412 (Europe) and M5938 (USA) both contain genes with greater than 90 % amino acid identity to phage c2 gpl14 and greater than 79 % identity to phage c2 gpl16. bIL67-type phages M6162, M6165, M6202 and D4410 each contain genes with greater than 88 % amino acid identity to bIL67 gp34 and gp36. Amino acid and secondary structure prediction alignments also identify areas of difference between the homologues (Supplementary Files). Therefore, these genes may be involved with the requirement for *pip *or *yjaE*. gpl14/gp34 are classified as hypothetical proteins/unknown function (NP_043562/NP_042311). HHpred analyses of gpl14/gp34 from all phages in this study result in top domain hit 5E7T (5e7t_B); minor structural protein 5; bacteriophages; *Lactococcus lactis.* Minor structural protein 5 is identified as ORF 52/BppA in lactococcal phage TUC2009 (accession number NC_002703), a component of the tripod baseplate structure with a putative carbohydrate binding domain and involved in receptor binding (McGrath *et al.*, 2006, Legrand *et al.*, 2016). gpl16/gp36 are classified as minor structural/hypothetical proteins (NP_043564/NP_042309), possibly collar proteins as a gene with 76 % identity to ORF36 was found in addition to *l16* in a phage c2 isolate exhibiting a prominent collar structure at its head–tail interface (accession number AAD20610). HHpred analyses of gpl16/gp36 from all phages in this study likewise identified carbohydrate binding domains with top hits to 1GU3 and 1GU1. McGrath *et al*. (2006) showed that the saccharide binding site of Tuc2009 Bpp A is more similar to 1GU1 in structural comparisons.

gpl15/gp35 is a minor structural protein proposed to be involved in phage adsorption to the host (NP_043563/ AAO49840) (Stuer-Lauridsen *et al.*, 2003). As with gpl14/gp34, HHpred analyses of all phages, except M6162, gave a top domain hit to 5E7T (5e7t_B). It has been previously reported that the N termini of gpl15/gp35 are conserved, the C termini less conserved, and the middle region variable and probably responsible for host recognition (Stuer- Lauridsen *et al.*, 2003). With regards to gpl15/gp35, the N-terminal 98 amino acid residues in each of the industrial phages studied here are indeed conserved with the N-terminal 98 residues of c2 and the N-terminal 101 residues of bIL67 (91.4 % pairwise amino acid identity). The C-terminal end (the last 76 amino acid residues for bIL67-type phages and the last 65 residues for c2-type phages) is more divergent (63.3 %) when all the *C2viruses* in this study are compared but shares more identity within a type: 88.7 % for c2-type (D4412, M5938 and c2) and 84.3 % for bIL67-type (D4410, M6162, M6165, M6202 and bIL67). Based on this data the C-terminal end of gpl15/gp35 could play a role in the requirement for *pip *or *yjaE*.

The region of gpl15/gp35 between the conserved N-terminal and C-terminal regions was previously shown to be variable and involved in host recognition for *C2viruses *bIL67 and CHL92 (Stuer-Lauridsen *et al.*, 2003). Recombinant bIL67 isolates which maintained the 5′ and 3′ ends of gene ORF35 but exchanged the middle section for that of phage CHL92 ORF2 (ORF35 homologue) acquired the host range and adsorption patterns of CHL92. The middle regions of gpl15 homologues in c2-type phages D4412 and M5938 and gp35 homologues in bIL67-type phages M6165 and D4410 share 90 % pairwise amino acid identity, and are 100 % identical between M5938 and M6165 (see also Supplementary Files). While this comparison would support the involvement of the gpl15/gp35 middle section in host-range determination, bIL67-type phages M6162 and M6202, which were not included in this alignment, suggest the participation of other factors. For M6162, there are only 2 aa between the gp35 conserved N-terminal and C-terminal ends and notably the 5e7t_B domain is absent. For M6202 the middle region of gp35 shares no identity with those of the other phages, but does retain the 5e7t_B domain. Nevertheless, M6162 adsorbs to DGCC7271 at 98 % which is equivalent to phages M6165, M5938 and D4412, which contain the conserved middle region. M6202 adsorbs just slightly less efficiently at 91 % (Table 3). This suggests that host recognition may be independent of the gpl15/gp35 middle region in this phage–host system or the different phage may be targeting the same host using different recognition sites. It is also possible that multiple middle regions of gpl15/gp35 can allow for recognition of the same primary receptor.

**Table 3.** Comparison of adsorption and efficiency of plaquing (EOP) of select phages on DGCC7271 and IL1403

	DGCC7271		IL1403	
Phage	Percentage adsorption	EOP	Percentage adsorption	EOP
M6162	97.7±0.4	1	–	–
M6165	98.0±0.6	1	–	–
M6202	91.0±4.0	1	–	–
D4412	98.2±1.4	1	–	–
M5938	98.5±0.9	1	51.0±13	1.5×10^−^^4^
bIL67	67.3±8.0	<1×10^−8^	62.6±6.8	1
M6653	96.3±2.5	1	60.9±11	9.2×10^−^^2^
M6654	97.5±1.7	1	67.0±4.3	8.2×10^−2^

(–) Not tested.

Outside of the ‘variable region’ of *l14–l15–l16* or ORF34–35–36, the European phages D4412 (c2-type) and D4410 (bIL67-type) are 98.5 % identical at the nucleotide level while the US phages M5938 (c2-type) and M6162, M6165 and M6202 (bIL67-type) share 97.9 % pairwise nucleotide identity. c2-type phages D4412 (European isolate) and M5938 (US isolate) share 89.8 % nucleotide identity outside of the ‘variable' region. bIL67-type phages D4410 (European isolate) and M6165, which of the North American phages shares the most overall identity with D4410, are 89.8 % identical at the nucleotide level outside of the variable region. It is interesting to speculate if this slight difference in similarity is a result of geographical separation and/or reflects the isolation of the European phages being 7 years earlier.

### Hybrid c2-type phage

To further study the relationship of the lactococcal host *pip *and* yjaE *genes with phage encoded *l14–l15–l16 *and ORF34–35–36 we attempted to exchange *l14–l15–l16 *with ORF34–35–36 in M5838 based on a method described by Rakonjac *et al.* (2005) in which recombinants of *C2viruses* c2 and 923 were isolated. Lubbers *et al.* (1995) had shown that ORF31/*l10* in *C2like viruses* encode a putative tail adsorption protein that may be involved in host-range determination. Rakonjac *et al*. (2005) further showed *l10* to be involved in DNA entry into the host. While bIL67 does not form visible plaques on DGCC7271, it adsorbed at 67 % efficiency which is similar to adsorption levels reported on its homologous host IL1403 of 26 % (Stuer-Lauridsen *et al.*, 2003) and 63 % in our experiments. Based on the high degree of similarity between bIL67 ORF31 and the homologues of DGCC7271 infective phages (93 % average protein identity), bIL67 may still be capable of injecting its DNA into the cell, enabling recombination with co-infecting phage. In support of this hypothesis, we tested M5938 infection on IL1403 where we observed an adsorption rate of 51 % and a reduced plaquing efficiency of 1.5×10^–4^ (Table 3).

DGCC7271 was propagated in broth to an OD_600_ of approximately 0.25 then co-infected with approximately 1×10^8^ p.f.u. ml^−1^ of phages M5938 and bIL67. After complete lysis of the culture, 100 µl of the filter-sterilized lysate was spotted onto *pip *mutant DGCC11032, which generated plaques. A set of 27 plaques was isolated and propagated on DGCC11032. To confirm the identity of the recombinant phages, PCR reactions were designed spanning four regions within the first 8 kb where deletions occur in the alignment of the genomes of bIL67 and M5938 (data not shown) thereby providing a marker of phage origin. Amplicon lengths were consistent with M5938 for all 27 isolates in three out of four regions spanning the 3′ end of the 8 kb segment. Nine of the phage isolates produced amplicons consistent with bIL67, and 18 produced amplicons consistent with M5938 in 5′ region. This indicated that at least two different recombinants had been obtained. The genomes of one representative isolate of each type, designated M6653 and M6654, were sequenced. The 22 157 bp genome of M6653 is 100 % identical to M5938 in three regions (nucleotides 1–7537, 9590–16083 and 22141–22157). Nucleotides 7637–9597 and 16049–22157 are 100 % identical to bIL67 (note overlapping identities). As shown in Fig. 1, the mosaicism of phage M6653 as compared with M5938 and bIL67 suggests that multiple recombination events resulted in its creation. Recombinant phage M6654 (22 350 nt) is 100 % identical to M5938 from nucleotides 1198–16825 with the remainder being 100 % identical to bIL67 (nucleotides 1–1242/16821–22350, note overlapping identities) indicating a single recombination event. For both M6653 and M6654, genes ORF34–35–36 from bIL67 were present rather than *l14–l15–l16 *homologues from M5938 which is consistent with their ability to infect the *pip* mutant DGCC11032 (Fig. 1). Concomitantly, M6653 and M6654 were unable to infect *yjaE *mutant DGCC11785. This substitution of the resident *l14–l15–l16 *genes with ORF34–ORF35–ORF36 further confirms that one or all of these genes contribute to the requirement for *pip *or *yjaE*. This data also corroborates this diverse region being a structural module for phage evolution.

Recombinant phages M6653 and M6654 are capable of infecting DGCC7271 despite the acquisition of bIL67 genes *34–35–36*. When tested, M6653 and M6654 adsorbed to DGCC7271 at 96–98 %, equivalently to M5938 and better than bIL67 (67 %) (Table 3). This result contrasts with those of Stuer-Lauridsen *et al.* (2003) where when the middle section of bIL67 ORF35 was exchanged for the middle section of ORF2 (ORF35 homologue) from phage CHL92, the adsorption phenotype adopted that of CHL92. As noted from the broader comparison of the DGCC7271 phages, the middle section of the respective gp35/gpl15 proteins does not appear to be the driving host-determination factor for this host. Additionally, M6653 and M6654 were found to plaque on IL1403 approximately three orders of magnitude better compared with M5938 (Table 3). This may be attributable to phage and/or host-encoded factors related to the infection process or to host defensive mechanisms and bears further study.

### *In vivo* genetic exchange

c2-type phage M5938 and bIL67-type phage M6162 genomes are 99.98 % identical at the nucleotide level over the first 17.6 kb but only 51.9 % identical in the ‘variable region’ suggesting genetic exchange with wild-type phages. Their subsequent prevalence in the North American industrial setting is undoubtedly in response to the introduction of the *pip* mutant DGCC11032. We were able to observe a similar genetic exchange between M5938 and bIL67 when co-infecting DGCC7271 with the two phages. Additionally, the European phages D4410 (bIL67) and D4412 (c2) are more similar to each other outside of the ‘variable region’ than they are to their respective *C2viruses *isolated in the USA. Similarly, the sequenced North American bIL67-type and c2-type (M5938 only) phages are more similar to each other outside of the ‘variable region’ than they are to European phages D4410 and D4412, respectively. This suggests that separate recombination events occurred in Europe and in North America.

### Application and further direction

Based on our data, there is a clear correlation between the region encoding *l14–15–16 *(c2) or ORF34–35–36 (bIL67) and use of the Pip or YjaE, respectively. Amino acid and predicted secondary structure alignments give indications as to differences between the respective proteins that serve as a basis for future investigation (Supplementary File). Additional study of lactococcal phage–host systems in the DuPont collection have verified this correlation (data not shown). A survey of over 100 *C2viruses* in the DuPont collection shows that each phage contains one set of these genes or the other (data not shown). This suggests that the three respective genes in each set may function together and that the corresponding gene from one set cannot function in place of its counterpart in the other set. Further study is needed to confirm this and to fully define the roles of each gene.

For additional insight, compare lactococcal *C2viruses* with one of the most extensively studied Gram-positive host–phage interactions between phage SPP1 and *B. subtilis. *The *B. subtilis *genome encodes the membrane-bound protein YueB which is an orthologue of Pip. Similar to *C2viruses*, phage SPP1 first binds reversibly to carbohydrate moieties, specifically the cell-wall teichoic acids, then, as a precursor to phage DNA ejection, irreversibly to *B. subtilis* YueB (Plisson *et al.*, 2007; Vinga *et al.*, 2012). Note that as YjaE and Pip are structurally similar (HHpred analysis identified domains 3CNI and 2CH7 in both), the same inference can be extended to both proteins.

Structural studies identified SPP1 tail spike protein gp21, specifically its C terminus, binding to YueB (São-José *et* *al*., 2004, 2006). The N erminus of SPP1 gp21 shares 26 % amino acid identity to the N terminus of lactococcal phage tail proteins, Tal_2009_ (Tuc2009 ORF53) and Tal_901-1_ (TP901-1 ORF47) (Plisson *et al.*, 2007; Stockdale *et al.*, 2013). Taxonomically, Tuc2009 and TP901.1 belong to the polythetic P335 lactococcal phage group which is morphologically and genetically distinct from *C2viruses *(Deveau *et al.*, 2006) and have been shown to not require Pip for infection (Krauss & Geller, 1998). Structurally, HHpred predicts a phage structural and carbohydrate binding domain, but does not identify a conserved domain in the C terminus SPP1 gp21. Although our results show a clear relationship between gpl14–15–16 and gp34–35–36, with their corresponding YueB orthologues Pip and YjaE, respectively, structural domain analysis does not predict direct protein interaction. Rather gpl14–15–16 and gp34–35–36 are more similar to Tuc2009 BppA Tuc2009 for which the exact function in host adsorption is yet to be fully elucidated (Legrand *et al.*, 2016).

Phage genome analyses show evidence of modular genetic exchange which we then demonstrated by *in vivo *creation of hybrids between phages bIL67 and M5938. These results show that host-determinant specificity serves as strong selective pressure for phage evolution via recombination. Subsequently, mutants with both a *pip *and a *yjaE *mutation were isolated from a single high-titre challenge of DGCC11032 with phage M6162. A representative double mutant, designated DGCC11572, was confirmed to harbour the same *pip *deletion as DCGG11032 and an additional mutation in *yjaE* consisting of a C>T transition at nucleotide 958 creating a TAA stop codon that would result in a putative 319 aa truncated protein. DGCC11572 is fully resistant to all *C2viruses* tested in this study (Table 1). Therefore, mutation of both *pip *and *yjaE* would be the most effective strategy to enhance phage robustness of commercially important starter strains and eliminate *C2viruses* from the industrial setting.

## Methods

### Bacteria, bacteriophages and culturing conditions.

Bacterial strains and bacteriophages are listed in Table 2. All *L. lactis* strains were grown at 30 °C in sterile 11 % nonfat dry milk or in M17 broth (Becton Dickinson) supplemented with 0.5 % lactose (all strains except IL1403) or glucose (IL1403). Preparation of bacteriophage lysates and standard plaque assays were performed as described by Terzaghi & Sandine (1975). Single-plaque isolates were purified from commercial whey samples and high-titre lysates passed through a 0.45  µm filter and stored at 4 °C. Phages M5938, M5939 and M5940 were isolated at approximately the same time period in 2010 from three distinct geographical locations in the USA while D4410 and D4412 were isolated in Europe in 2003. Phages M6162, M6165 and M6202 were isolated from industrial whey samples from three distinct geographical locations in the USA and Canada in 2012. Phage typing was performed using the multiplex PCR method as described by Labrie & Moineau (2000). Adsorption assays were performed as described by Sanders & Klaenhammer (1980).

### Generation of bacteriophage-insensitive mutants (BIMs).

High-titre phage lysates of phage M5938, M5939 and M5940 were combined into a three-phage cocktail. A single-step phage challenge was performed by exposing DGCC7271 to this phage cocktail at varying m.o.i. Separate single-step challenges were performed on DGCC7271 with phages M6162 and M6165 at varying m.o.i. Phage-resistant survivors were tested via PCR and sequencing for disruption of the *pip* or *yjaE *genes. For isolation of double mutants, DGCC11032 was challenged with high-titre phage lysate of M6162 at varying m.o.i.

### PCR, DNA preparation, restriction digests, sequencing and *in silico* analysis.

PCR was performed with GoTaq^®^ Green Master Mix (Promega) or Phusion HF Mastermix (New England Biolabs) according to the manufacturer’s instructions. Primers were synthesized by Integrated DNA Technologies (Coralville, IA, USA), and primer sequences are listed in Table 2. PCR products were prepared for sequencing using a QIAquick PCR Purification Kit (Qiagen). Sequencing of PCR amplicons was performed by Eurofins MWG Operon (Huntsville, AL, USA), and results were analysed using Clustal Omega (Sievers *et al.*, 2011). Primers derived from the MG1363 genome (NC009004) or the IL1403 genome (AE005176) were used to analyse the *pip* gene. DGCC7271 and BIM (bacteriophage-insensitive mutant) *pip* genes were amplified and sequenced with primers pipStartF and pipEndR. Primers derived from the IL1403 genome were used to analyse the *yjaE* gene. *yjaE* sequences from DGCC7271 and BIMs were amplified with YjaEF1 and YjaER. Amplicons were sequenced with YjaEF1, YjaEF2, YjaEF3, YjaER and the reverse complements of YjaEF2 and YjaEF3. Phage DNA was isolated from high-titre phage lysate using Invitrogen PureLink Viral RNA/DNA Mini kit (Life Technologies) according to manufacturer’s instructions. Eluted RNA/DNA was treated with RNaseA (Thermo Scientific) at a concentration of ~0.5 µg µl^−1^.

### Phage genome sequencing.

For all phages sequenced in this study except M6653 and M6654, the Invitrogen Quant-iT Picogreen dsDNA Assay Kit (Life Technologies) was used to determine concentration of phage DNA. Phage DNA was diluted to 1 ng in 5 µl H_2_O. Library preparation was performed using the Illumina Nextera^®^ XT DNA Sample Preparation Kit. The DNA library was then sequenced with the MiSeq Reagent Kit V2 on a MiSeq system (Illumina). Ray Meta was used for genome assembly (Boisvert *et al.*, 2012).

For phages M6653 and M6654, the genomic DNA was prepared according to a library construction protocol developed by Illumina and sequenced using the Illumina MiSeq. Briefly, after genomic DNA was sheared with a Covaris S220 instrument, the resulting DNA fragments were end-repaired and their 3′ ends treated for A-base addition. After ligation of Illumina-specific adapters and gel-based size-selection, adapter-ligated DNA fragments were subjected to limited PCR amplification with Illumina-specific PCR primers. Cluster generation and paired-end sequencing of the amplified DNA fragments were performed on an Illumina MiSeq, according to Illumina’s instructions. A single flow cell was loaded with the DNA fragments from the strain. Sequences and quality scores were generated with the Illumina pipeline software for image analysis and base calling. After initial base calling and processing, the sequencing files generated by the Illumina pipeline were converted to FASTQ format and additional custom quality filtering was performed, such that reads were trimmed if they harboured one or more base at their 3′ end with a quality score of less than 15. Quality-trimmed Illumina reads were assembled into contigs using the SPAdes assembler (v3.0.0) with default parameters (Bankevich *et al.*, 2012). The SPAdes module to perform error correction of small SNPs/INDELs in the contigs was also run. Contigs greater than 500 bp in length with coverage greater than two (as computed by SPAdes) were considered for downstream analysis. Genome annotations were performed using RAST (Aziz *et al.*, 2008; Overbeek *et al.*, 2014) and alignments were performed using ClustalW default parameters within Geneious (Geneious version 6.1.4, http://www.geneious.com/) (Kearse *et al.*, 2012). Protein secondary structure analysis was performed using the HHpred Bioinformatics Toolkit (Söding *et al.*, 2005).

## Supplementary Data

1014 Supplementary File 1
