# Supplementary material for: Genetic determinants of lactococcal C2viruses for host infection and their role in phage evolution
Source: J Gen Virol. 2016 Aug;97(8):1998–2007. doi: 10.1099/jgv.0.000499 (PMC5156332; doi:10.1099/jgv.0.000499)

Supplementary File: gp114-l15-16 and gp34-35-36 amino acid percent identity and amino acid conservation and secondary structure alignment.

Percent amino acid identity was performed using ClustalW default parameters within Geneious (Geneious version 6.1.4, http://www.geneious.com/) (Kearse et al., 2012). For gpl15 / gp35, the respective proteins were divided into N-terminal, middle, and C-terminal for identity determinations. Amino acid conservation and secondary structure prediction alignment was performed using PRALINE Multiple Sequence Alignment set at default parameters (http://www.ibi.vu.nl/programs/pralinewww/) (Simossis & Heringa, 2005). The color key for the amino acid conservation output is as shown. Alignment comparison of predicted secondary structure used DSSP (Kabsch & Sander, 1983) and PSIPRED (Jones, 1999) and was performed separately for each c2-type (gpl14-l15-l16) and bIL67-type (gp34-35-36) group of phage proteins; color assignments are red for helix and blue for sheet.

Jones, D.T. (1999). Protein secondary structure prediction based on position-specific scoring matrices. *J Molec Biol* **292**,195-202.

Kabsch, W. & Sander, C. (1983). Dictionary of protein secondary structure: pattern recognition of hydrogen-bonded and geometrical features. *Biopolymers* **22**, 2577-637.

Kearse, M., Moir, R., Wilson, A., Stones-Havas, S., Cheung, M., Sturrock, S., Buxton, S., Cooper, A., Markowitz, S. & other authors (2012). Geneious Basic: an integrated and extendable desktop software platform for the organization and analysis of sequence data. *Bioinformatics* **28**, 1647-1649.

Simossis, V.A. & Heringa, J. (2005). PRALINE: a multiple sequence alignment toolbox that integrates homology-extended and secondary structure information. *Nucleic Acids Res* **33** (Web Server issue), W289–W294.

gpl14 – gp34 amino acid alignment / % identity

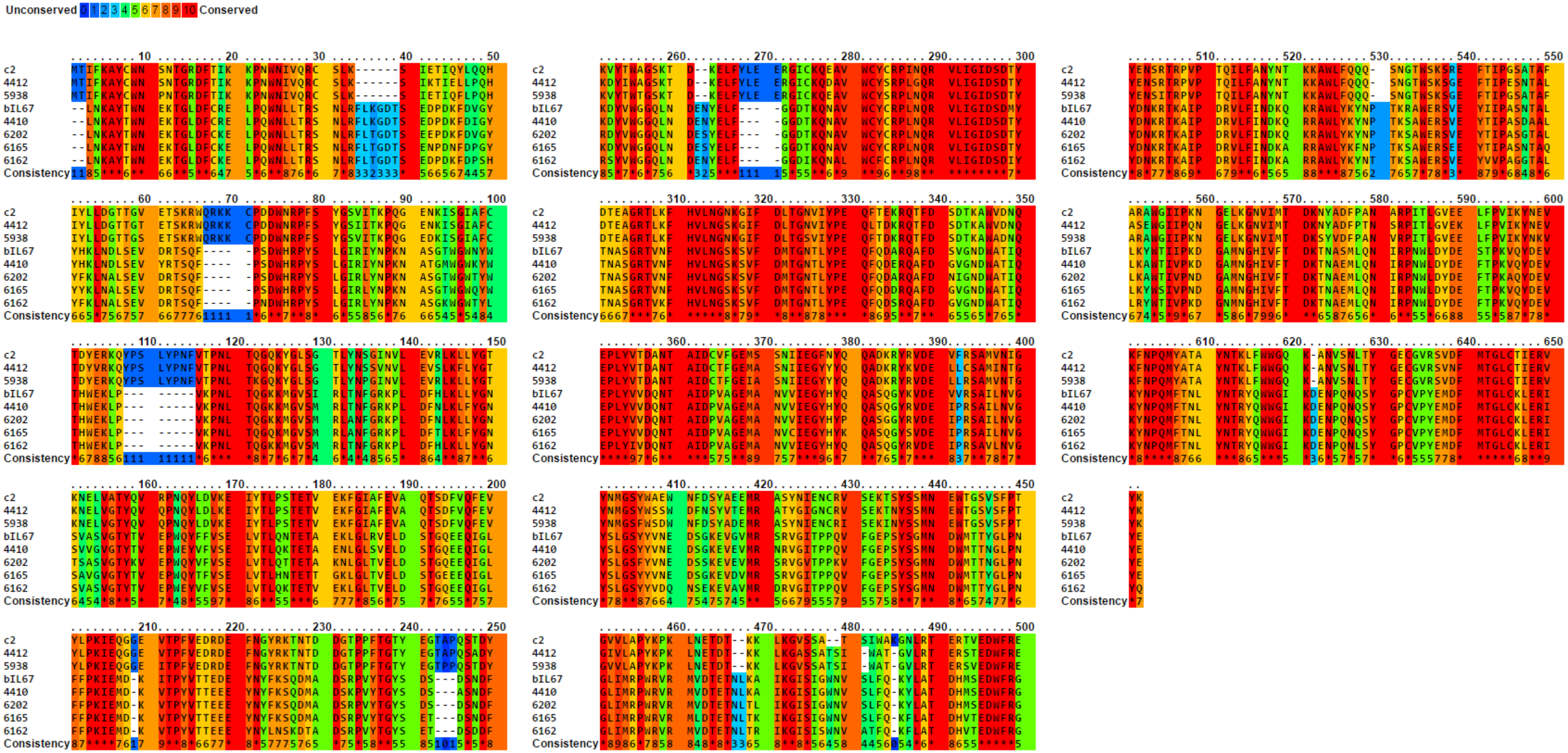

	c2	D4412	M5938	bIL67	D4410	M6162	M6165	M6202
c2		90	94	42	42	41	41	41
D4412	90		91	42	41	40	41	41
M5938	94	91		41	41	41	41	40
bIL67	42	42	41		94	89	91	93
D4410	42	41	41	94		89	92	93
M6162	41	40	41	89	89		89	89
M6165	41	41	41	91	92	89		92
M6202	41	40	40	93	93	89	92	

gpl14 secondary structure prediction

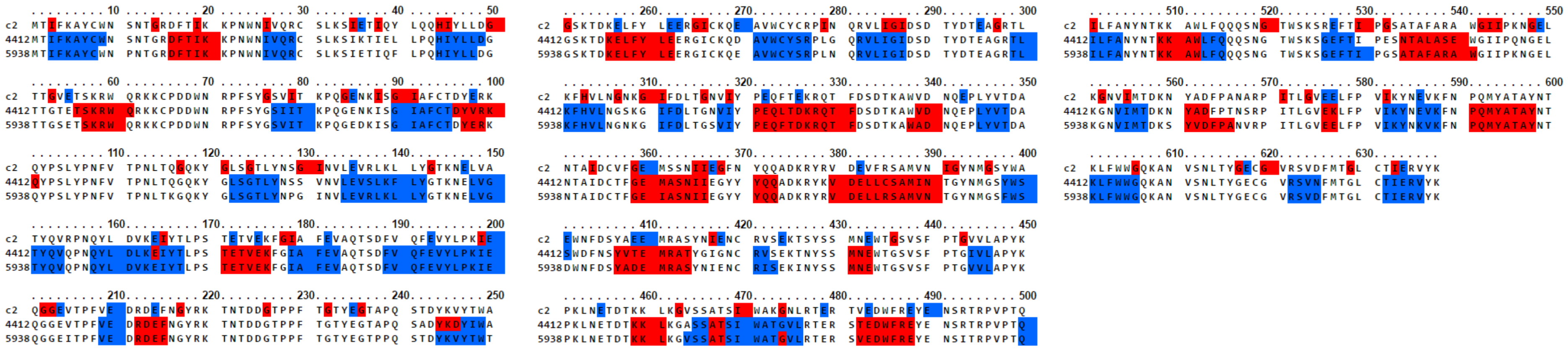

gp34 secondary structure prediction

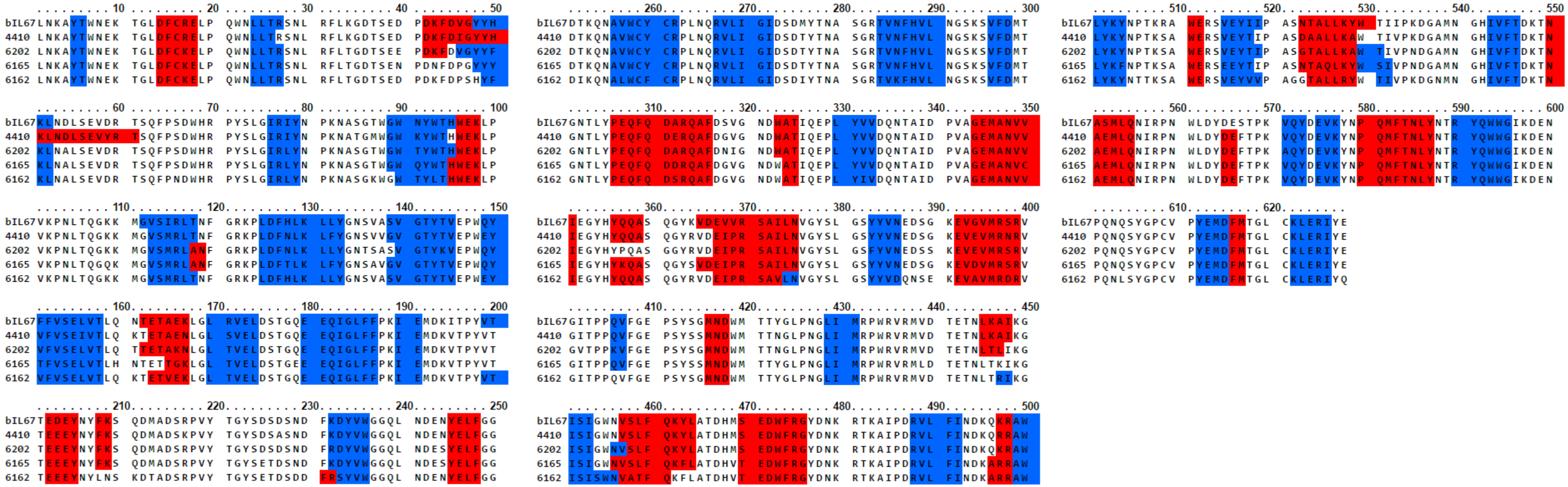

gpl15 – gp35 amino acid alignment / % identity

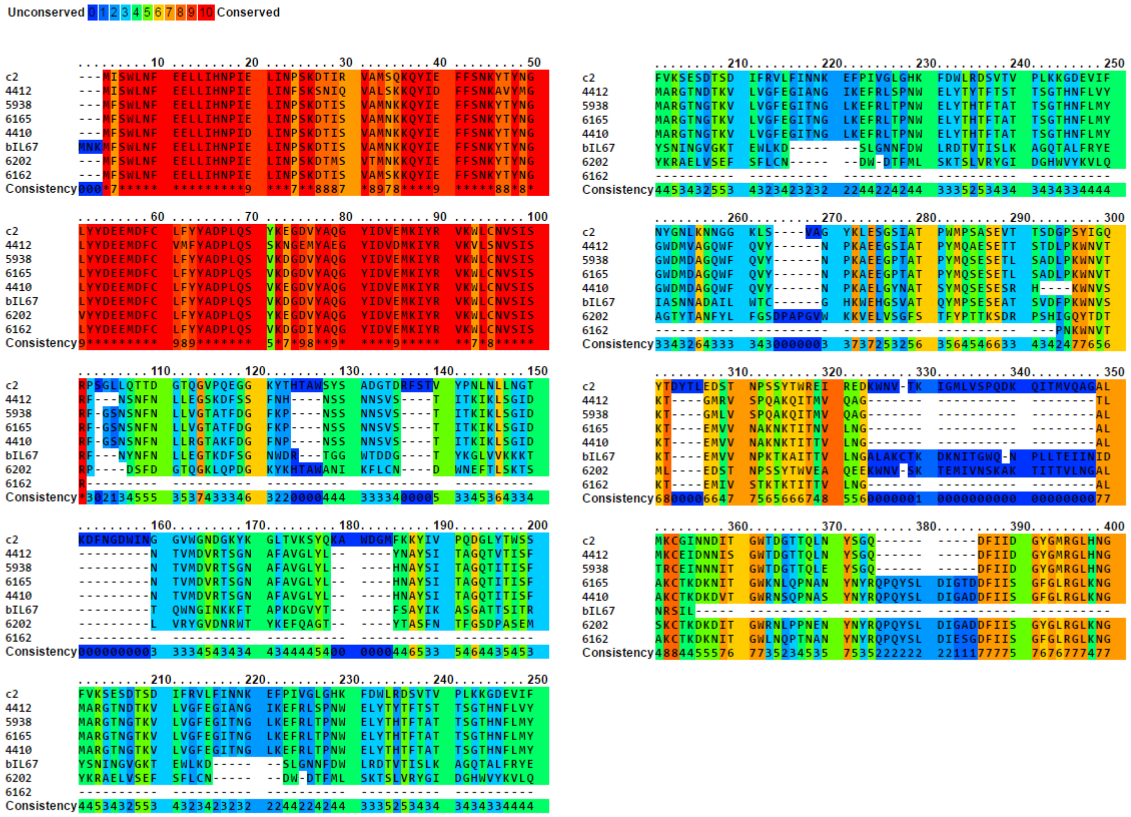

gpl15 / gp35 N-terminal

	c2	D4412	M5938	bIL67	D4410	M6162	M6165	M6202
c2		79	94	94	94	94	94	93
D4412	79		78	78	77	79	78	75
M5938	94	78		100	98	98	100	95
bIL67	94	78	100		98	98	100	95
D4410	94	77	98	98		96	98	95
M6162	94	79	98	98	96		98	93
M6165	94	78	100	100	98	98		95
M6202	93	75	95	95	95	93	95	

gpl15 / gp35 Middle

	c2	D4412	M5938	bIL67	D4410	M6162	M6165	M6202
c2		22	25	0	24	-	25	0
D4412	22		85	28	84	-	85	0
M5938	20	85			94	-	100	0
bIL67	45	28	25		24	-	25	0
D4410	19	84	94	24		-	94	0
M6162	-	-	-	-	-		-	-
M6165	-	85	100	25	94	-		0
M6202	8	0	0	0	0	-	0	

gpl15 / gp35 C-terminal

	c2	D4412	M5938	bIL67	D4410	M6162	M6165	M6202
c2		88	89	45	42	47	45	46
D4412	88	89	89	46	41	49	46	46
M5938	89	89		45	41	47	45	45
bIL67	45	46	45		86	82	86	84
D4410	42	41	41	86		80	90	87
M6162	47	49	47	82	80		84	82
M6165	45	46	45	86	90	84		84
M6202	46	46	45	84	87	82	84	

gpl15 secondary structure prediction

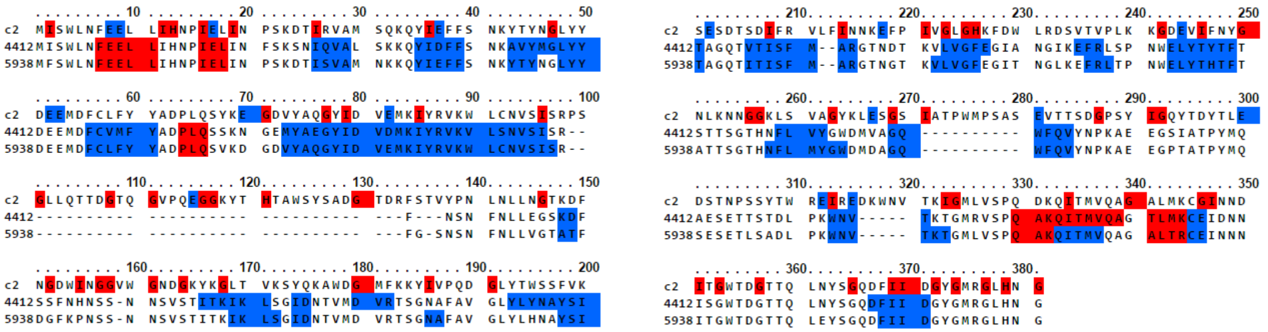

gp35 secondary structure prediction

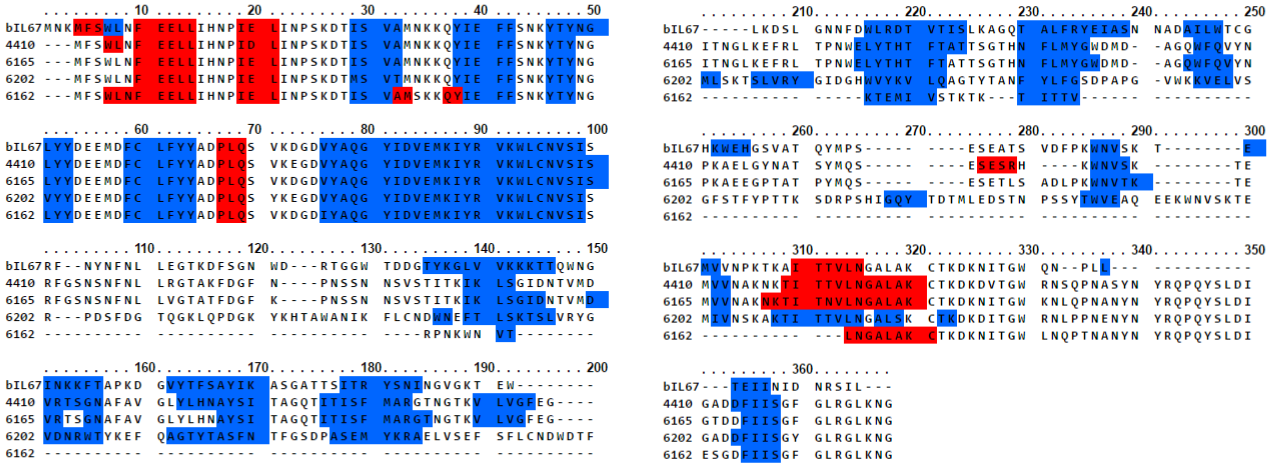

gpl16 – gp36 amino acid alignment / % identity

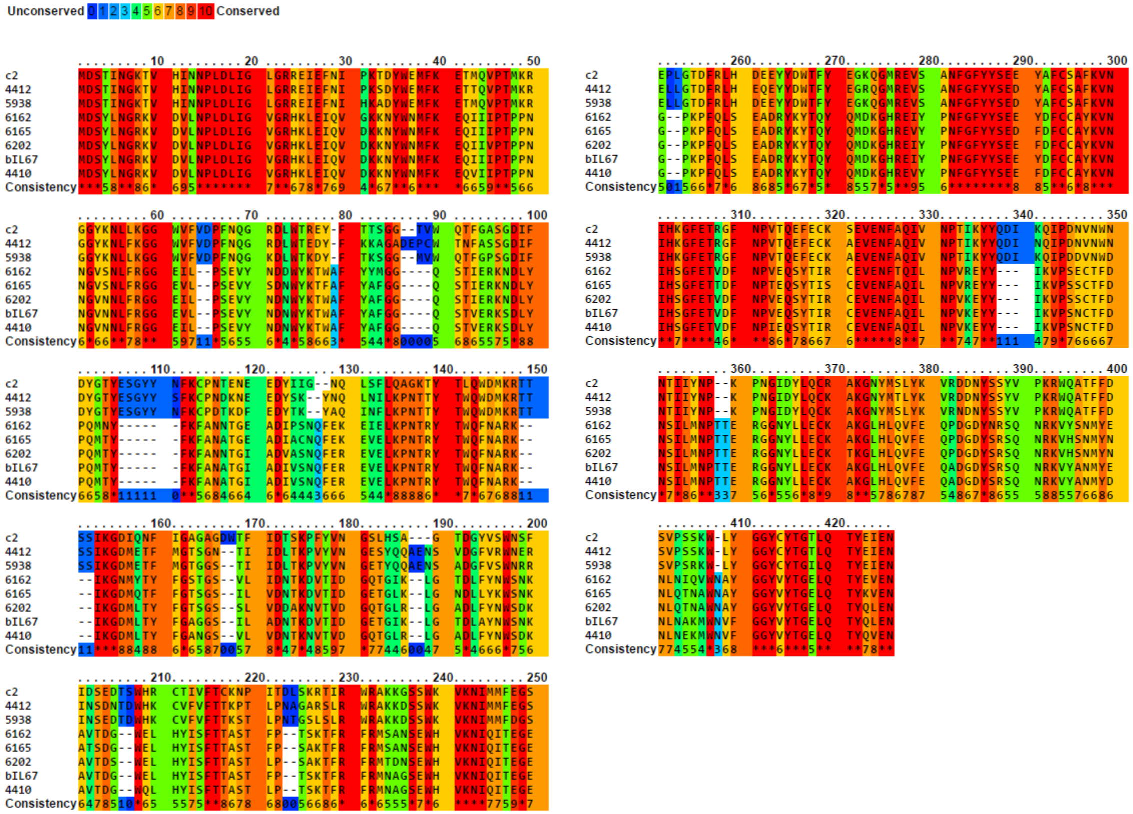

	c2	D4412	M5938	bIL67	D4410	M6162	M6165	M6202
c2		79	81	38	37	37	37	37
D4412	79		88	39	39	38	40	39
M5938	81	88		40	39	39	40	39
bIL67	38	39	40		94	90	88	89
D4410	37	40	40	94		89	88	91
M6162	37	38	39	90	89		89	88
M6165	37	40	40	88	88	89		90
M6202	37	39	39	89	91	88	90	

gpl16 secondary structure prediction

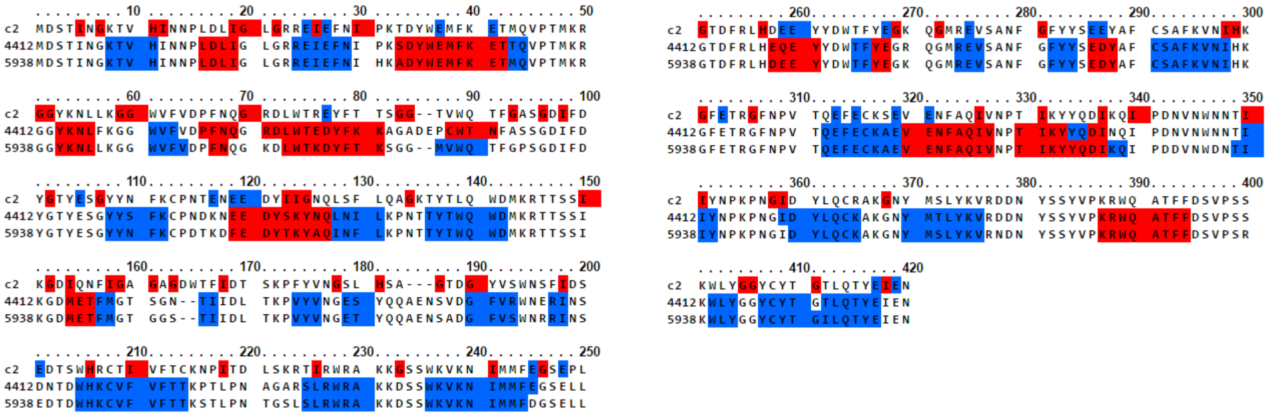

gp36 secondary structure prediction

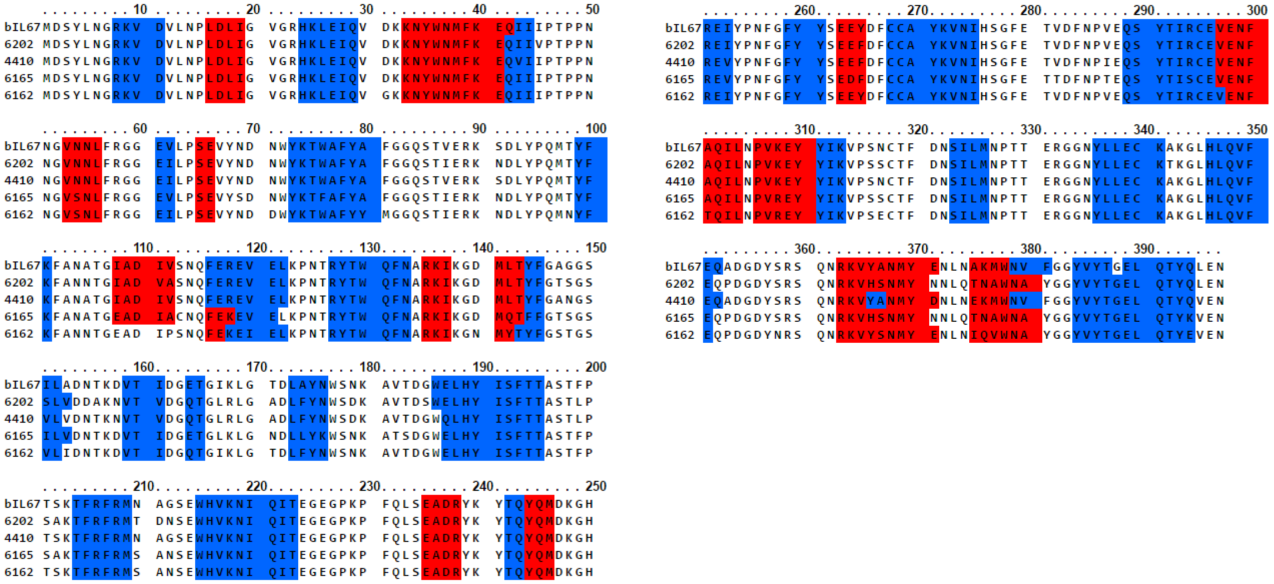

Supplement: Supplementary file 1 [file jgv-97-1998-s001.pdf]
